# Electroacupuncture for post-thoracotomy pain: A systematic review and meta-analysis

**DOI:** 10.1371/journal.pone.0254093

**Published:** 2021-07-07

**Authors:** Sohyeon Park, Yee Ran Lyu, So Jung Park, Min Seok Oh, In Chul Jung, Eun-Jung Lee

**Affiliations:** 1 College of Korean Medicine, Daejeon University, Daejeon, Republic of Korea; 2 Clinical Trial Center, Dunsan Korean Medicine Hospital of Daejeon University, Daejeon, Republic of Korea; 3 Department of Korean Internal Medicine, College of Korean Medicine, Daejeon University, Daejeon, Republic of Korea; 4 Department of Korean Rehabilitation Medicine, College of Korean Medicine, Daejeon University, Daejeon, Republic of Korea; 5 Department of Oriental Neuropsychiatry, College of Korean Medicine, Daejeon University, Daejeon, Republic of Korea; University of Oxford, UNITED KINGDOM

## Abstract

**Background:**

Thoracotomy is an invasive surgical procedure that produces intense postoperative pain. Electroacupuncture has been used to induce analgesia in various situations, including after surgery. The aim of the following systematic review and meta-analysis was to evaluate the effect of electroacupuncture on post-thoracotomy pain.

**Methods:**

The studies for the systematic review were searched using the following 9 databases: PubMed, Cochrane Library, EMBASE, MEDLINE Complete, Google Scholar, China National Knowledge Infrastructure (CNKI), Korean Medical Database (KMBASE), Koreanstudies Information Service System (KISS), and OASIS, without language restriction. Randomized controlled trials (RCTs) that met the inclusion criteria were selected. The quality assessment was performed using the Cochrane risk-of-bias tool, and RevMan 5.3 was used for meta-analysis. The review protocol is registered in the International Prospective Register of Systematic Reviews (PROSPERO) as CRD42019142157.

**Results:**

Eleven randomized controlled trials were included in the systematic review. The meta-analysis was performed for two outcome measures: pain score 24 hours after surgery and total dose of opioid analgesics. A subgroup analysis was performed according to the control group: sham acupuncture and conventional analgesia group. Pain score 24 hours after surgery of electroacupuncture group showed a standard mean difference of -0.98 (95% CI: -1.62 to -0.35) compared to sham acupuncture. The standard mean difference was -0.94 (95% CI: -1.33 to -0.55) compared to conventional analgesia. The total dose of opioid analgesics of electroacupuncture group showed a standard mean difference values of -0.95 (95% CI: -1.42 to -0.47) compared to sham acupuncture. The standard mean difference was -1.96 (95% CI: -2.82 to -1.10) compared to conventional analgesia.

**Conclusion:**

Current evidence suggests that electroacupuncture might provide useful pain relieving effect on post-thoracotomy patients. However, due to low quality and high heterogeneity of existing data, further rigorously designed studies should be performed to confirm the safety and efficacy.

## Introduction

Thoracotomy is an invasive surgical procedure that damages the ribs, muscles, and peripheral nerves, leading to intense postoperative pain [1]. If the acute pain after surgery is inadequately addressed, it can lead to severe chronic pain [2]. Therefore, proper pain management is a major part of post-thoracotomy patient care and can have a huge influence on patients’ quality of life.

Various treatment methods are used to control pain after thoracotomy, such as epidural analgesia, peripheral nerve block, and systemic treatment [1]. In many cases, opioid treatment is the predominant option in patients with post-thoracotomy pain [1]. However, opioids have multiple potential adverse effects, such as respiratory depression, cough suppression, reduced intestinal motility, nausea, vomiting, and urinary retention [3]. They are also highly addictive: 64.4% (42,249) of deaths related to drug overdose in the United States in 2016 involved opioids, including prescription opioid analgesics [4]. Therefore, clinicians should seek to decrease the total required dose of opioids while managing pain after surgery.

Among the various treatment options, electroacupuncture (EA) has been successfully used to control pain after a variety of surgeries, including thoracotomy, lower abdominal surgery, and knee arthroplasty [5–7]. Several studies have attempted to elucidating the mechanism by which EA alleviates pain. According to these studies, when EA is performed, bioactive chemicals including opioids that block pain are released through peripheral, spinal, and supraspinal mechanisms [8–11]. In addition, many studies have suggested that EA provides effective pain management when combined with conventional analgesics, so it may reduce a patient’s chance of suffering the side effects of pharmaceuticals [8,12]. However, there are also few studies raising controversy on analgesic effect of EA [13,14].

With increasing demand for both effective postoperative pain management and evidence-based medicine, several systematic reviews and meta-analyses have investigated the use of EA to manage pain after different surgeries [15,16]. The objective of the present systematic review and meta-analysis was to evaluate the analgesic effects of EA in patients with post-thoracotomy pain.

## Methods

The present study was registered in the International Prospective Register of Systematic Reviews (PROSPERO) as CRD42019142157, and the protocol of the study was published [17]. The systematic review and meta-analysis followed the PRISMA checklist.

### Search strategy

The review question “Is EA combined analgesia effective in reducing post-thoracotomy pain compared to conventional analgesia (CA) without EA?” was made using the PICO form. The following major databases were searched: PubMed, Cochrane CENTRAL, EMBASE, MEDLINE, and Google Scholar. Several Chinese and Korean databases were also searched because of the characteristics of the intervention: as a Chinese database, CNKI was included. Additional databases from Korea were KMBASE, KISS, and OASIS.

Combinations of search words were framed within three major categories: thoracotomy, postoperative pain, and acupuncture. The following key terms were used in each category: ("thoracotomy" OR "thoracic surgery" OR "lobectomy" OR "pneumonectomy" OR “pneumectomy” OR "esophagectomy" OR "open heart surgery" OR "cardiac surgery”) AND (“pain” OR “postoperative” OR “perioperative” OR “anagesia” OR “analges*”) AND (“acupuncture” OR “acupressure” OR “acupoint” OR “acup*” OR “electroacupuncture”). The term (NOT “Video-assisted”) was also used to exclude trials with participants who underwent video-assisted thoracic surgeries. The search targeted studies with the key terms in their titles or abstracts, without language restrictions. Adjustments were made according to each database’s search system. Since Google Scholar only limits searches to specific fields (title, author, a particular journal, and date), limiting the search to both title and abstract was impossible. Only title search was performed in Google Scholar after the initial trial of searching without any limitation, which led to too many unrelated articles. The entire search process was finalized on August 07, 2020. Duplicates within the search results were identified and removed first with the software EndNote and they were all double-checked by P.S.H. The remaining papers were sorted by title and abstract. Full text articles were assessed for eligibility, and the final selections for data synthesis were determined. Studies that met all inclusion criteria were selected.

### Inclusion criteria

#### Types of studies

Only Randomized controlled trials (RCTs) were included in the present systematic review because they occupy a high level in the hierarchy of evidence. Case reports, case series, literature reviews, and uncontrolled trials were not included, nor were animal studies or ongoing studies. The study inclusion and search were performed without language restriction. However, since the combinations of search words were in English, all searched items had at least English titles and abstracts, even if some of them were not written in English.

#### Types of participants

Patients who had undergone thoracotomy were included, without any restrictions on age, sex, ethnicity, type of surgery, or disease.

#### Types of interventions

EA was the intervention of all included studies. Other types of acupuncture without electrical stimulation were excluded.

#### Types of controls

Control group of the included studies was sham acupuncture group or CA group. In the studies that used sham acupuncture as the control, electrodes were connected to the acupuncture needles without stimulation. Sham transcutaneous electrical nerve stimulation was also included in this group since it basically uses as the same method as sham acupuncture. CA group had no treatment corresponding to EA.

#### Types of outcome measure

Any type of numerical pain score, including the Visual Analogue Scale (VAS), was considered as a primary outcome. The total amount of opioid analgesic, such as fentanyl, sufentanil, and morphine was considered as a secondary major outcome. These two outcomes were used in the quantitative data synthesis and meta-analysis. Other outcome measures, such as the amount of released chemicals with analgesic effects and the incidence of other postoperative complications were not included in the synthesis because not enough such data were available to synthesize.

### Data extraction

Two researchers (P.S.H. and L.E.J.) independently extracted data from selected studies. The extracted data included author, year of publication, study design, type of surgery, sample size (of each group), difference in baseline characteristics between intervention and control groups, treatments in the intervention group and control groups, outcomes, results, and conclusions. A third researcher (J.I.C.) confirmed the extracted data and resolved any discrepancies between the initial two researchers who performed the data extraction. The finalized information was summarized in a table and reviewed in a group discussion.

### Quality assessment

A quality assessment was performed independently by two researchers (P.S.H. and L.E.J.) using the Cochrane risk-of-bias tool in Cochrane Collaboration’s software RevMan 5.3 [18]. The tool contains the following seven domains: random sequence bias (selection bias), allocation concealment (selection bias), blinding of participants and personnel (performance bias), blinding of outcome assessment (detection bias), incomplete outcome data (attrition bias), selective reporting (reporting bias), and other biases [18]. The risk of bias was assessed at the study level. Discrepancies were discussed and resolved as a group, with input from a third researcher (J.I.C.).

The “GRADE profiler” program of the Cochrane Collaboration was used to assess the quality of the cumulative evidence. Grading of Recommendations Assessment, Development and Evaluation (GRADE) rates the strength of evidence in four levels: “Very low,” “Low,” “Moderate,” and “High” [19]. The quality of the accumulated evidence was assessed by evaluating risk of bias, inconsistency, indirectness, imprecision, and other considerations, as well as by labeling each category according to the GRADE guidelines with three labels: “Not serious,” “Serious,” and “Very serious.” By integrating the results of each category, the final rating of cumulative evidence strength was determined. The evidence strength was initially rated by two independent researchers (P.S.H. and L.E.J.) who filled in the GRADE profiler’s evidence table. A third researcher (J.I.C.) then reviewed and compared the two tables. Discrepancies were resolved in a group discussion, with professional statistical advice.

### Data synthesis

The software RevMan 5.3 was used for the meta-analysis. The meta-analysis was carried out according to the following two outcome measures: (1) pain score 24 hours after surgery and (2) total opioid analgesic dose. Both outcome measures in the meta-analysis were continuous and scales for assessing them were not all the same. Therefore, standardized mean difference (SMD) and 95% confidence interval (CI) were calculated using the extracted data (mean, standard deviation, and population size) to compare effect sizes. A random-effects model was chosen based on the different characteristics of the included studies, because the study results came from different population sizes and estimates of effect sizes. Statistical heterogeneity was estimated using the Higgins I^2^ test. I^2^ values of 25%, 50%, and were categorized as low, moderate, and high respectively [20]. Subgroup analysis was performed according to the type of control group: (1) sham acupuncture group (2) CA group.

A sensitivity analysis was performed to examine the accuracy of the decisions made in the meta-analysis. First, a fixed-effect model was tried out and mean differences were calculated to compare the results with those from the decided methodology and determine if the methodology was chosen appropriately. Second, the following method was performed to find out if any inclusion has affected the consistency of the meta-analysis. For the meta-analysis of *n* selected studies, new *n* meta-analyses were performed with only *n-1* studies, excluding a different study each time [21]. If heterogeneity significantly drops in a meta-analysis, the excluded study in the meta-analysis was dropped from the final meta-analysis. The synthesized results are summarized and illustrated visually using forest plots.

## Results

### Search result

**Fig 1:**
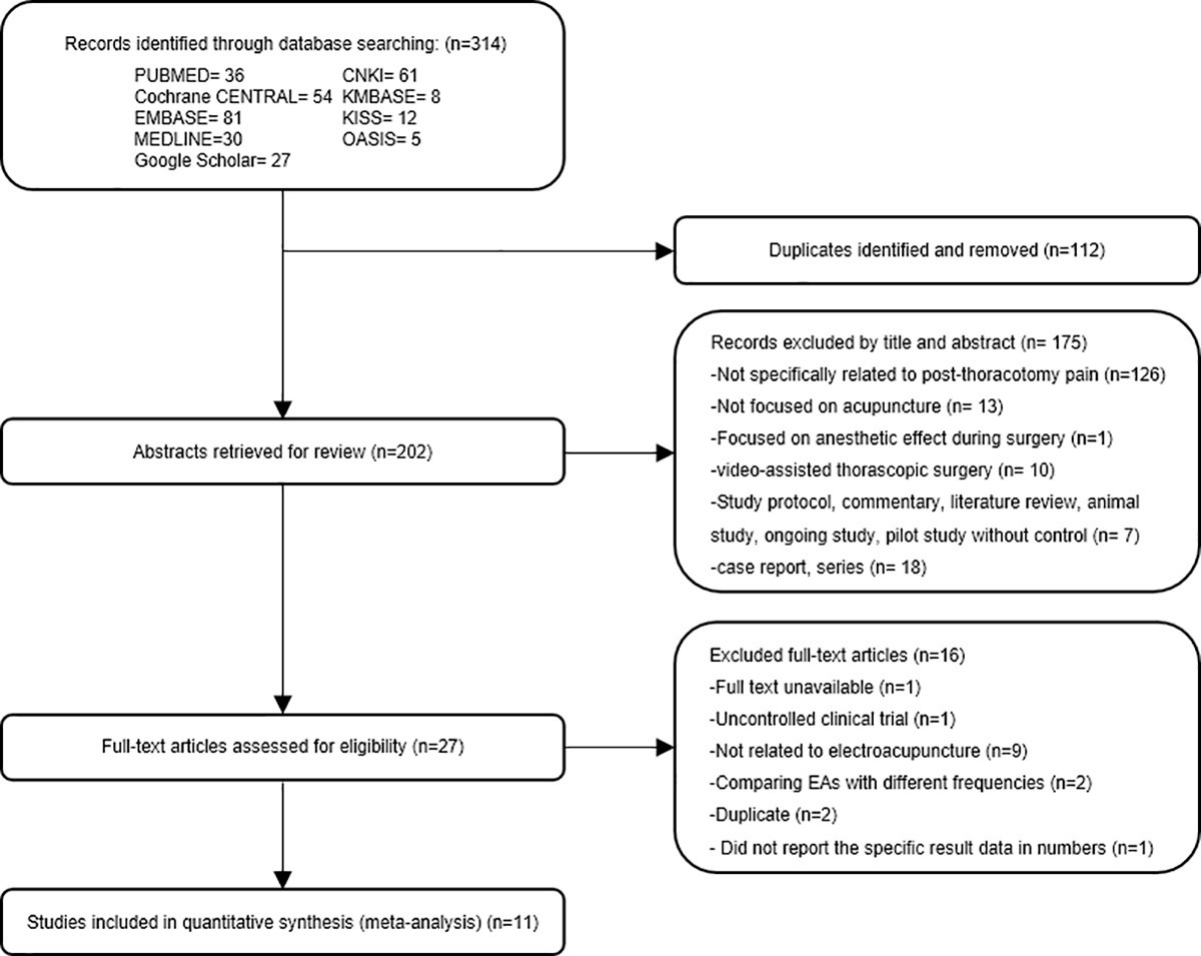
Flow diagram of the study selection process.

A total of 314 publications were initially identified by the search strategy across the 9 databases. After the removal of 112 duplicates, 202 papers were screened by titles and abstracts. A total of 175 papers were excluded because they (1) were not specifically related to post-thoracotomy pain, (2) were not focused on acupuncture, (3) focused on anesthetic effect during surgery, (4) included participants who underwent video-assisted thoracic surgery, and (5) had study designs that did not fit the inclusion criteria. During this step, all studies with abstracts that mentioned acupuncture interventions were included, even if EA was not specifically mentioned. They were retained for further full-text reading to check whether the specific process met the criteria for EA. The full texts of the remaining 27 studies were assessed for eligibility. Sixteen studies were subsequently excluded after full-text review, and 11 RCTs were finally included for review [5,22–31] (Fig 1).

### Description of included studies

A total of 11 RCTs met all the criteria and were ultimately included as sources of data for the present systematic review. Three of these trials compared EA group and sham group, seven compared EA group with CA group, and one compared EA group with both sham group and CA group (Table 1).

**Table 1:** Summary of included studies.

Author (year)	Study design	Participants			Intervention		Control	Outcomes
		Type of Surgery	Sample Size (A/B)	Baseline Characteristics Difference between Intervention and Control Groups				
Zhou (2017) [22]	RCT	Elective radical resection for esophageal cancer	60 (30/30)	No statistical difference (Age, body mass, operation time)	(A) EA	(B) sufentanil for PCIA		(1) total dosage of sufentanil (2) VAS (3) level of plasma β-EP, 5-HT and PGE_2_ (4) excellent analgesia rate (5) safety degree
Chen (2016) [23]	RCT	small incision lobectomy	92 (46/46)	No significant difference	(A) EA (1hr after surgery, repeated every 12hr for 3 days after surgery)	(B) Sham acupuncture		(1) VAS (2) postoperative use of sauteralgyl for the first 72 hrs (3) postoperative recovery data (incidence of nausea and vomiting, postoperative appearance of flatus and defecation (4) the blood levels of β-EP and 5-HT
Yu (2016) [24]	RCT	Cardiac surgery	50 (25/25)	No statistical difference	(A) Acupuncture combined with medicine (EA combined with injection of dexmedetomidine)	(B) conventional analgesia (dexmedetomidine)		(1) static and dynamic VAS (2) SAS scores (3) mean arterial pressure (4) heart rate (5) oxygen saturation (6) injection dosage of dexmedetomidine and morphine hydrochloride (7) analgesia and sedation satisfaction rate (8) incidence of adverse reactions (9) mechanical ventilation and ICU time, hospitalization expense
Wang (2015) [25]	RCT	elective lobectomy	80 (40/40)	No mentioned difference between the two groups	(A) electroacupuncture on the basis of general anesthesia	(B) general anesthesia		(1) VAS (2) total amount of sufentanil and dezocine (3) incidence rate of adverse reaction
Xie (2014) [26]	RCT	radical esophagectomy	60 (20/20/20)	No significant difference (operating time, the total amount of propofol and remifentanil)	(A) EAS	(B)Sham (C)general anesthesia		(1) VAS (2) Total amount of sufentanil (3) Plasma β-EP, 5-HT, and PGE_2_
Yu (2013) [27]	RCT	unilateral lobectomy	40 (20/20)	No mentioned difference	(A) electroacupuncture with PVB on the basis of PCA postoperatively	(B) PVB on the basis of PCA postoperatively		(1) total dosage of perioperative analgesics (sufentanil) (2) VAS (3) dosage of hemodynamic agents (4) dissipation time of nerve block (5) incidence of postoperative nausea and vomiting (6) time for extubation (7) incidences of respiratory complication
Zhao (2013) [28]	RCT	Lateral thoracotomy for esophageal cancer	120 (60/60)	No statistical difference	(A) EA	(B) medication (PCIA)		(1) VAS score (2) safety degree (3) β-EP level
Coura (2011) [29]	RCT	elective heart conventional surgery myocardial revascularization (8/8) valve replacement (5/1)	22 (13/9)	Demographic data is given. Age p-value 0.21	(A) preoperative EA at bilateral points and postoperative PCA	(B)sham TENS and postoperative PCA		(1) total dose of fentanyl (2) bolus dosage (3) bolus number (4) Verbal pain scale
Zhu (2011) [30]	RCT	lung-cancer thoracotomy ①benign lung lesion (6) ②malignant lung cancer (24) ③pulmonary lobectomy (24) ④total pneumonectomy (6)	120 (30/30/30/30)	No statistical difference	(A) preoperative acupuncture, intraoperative acupuncture, postoperative acupuncture analgesia (combined PCIA) (C) non-preoperative acupuncture, non-intraoperative acupuncture, and postoperative acupuncture analgesia (combined PCIA)	(B) preoperative acupuncture intraoperative acupuncture, and non-postoperative acupuncture (complete PCIA) (D) non-preoperative acupuncture, non-intraoperative acupuncture and non-postoperative acupuncture (complete PCIA)		(1) β-EP contents (2) cortisol contents (3) actual dose of Fentanyl after operation
Bachleda (2010) [31]	RCT	primary elective CABG or valve surgery	34 (16/18)	No significant difference (sex, age, NYHA, BMI, EF, ASA)	(A) EAA on the basis of standard anesthesia	(B) A continuous sufentanil infusion intraoperatively on the basis of standard anesthesia		(1) Duration MV (2) Sufentanil consumption (3) pritramid dosage after surgery (4) NAS
Wong (2006) [5]	RCT (double-blind, placebo controlled)	anatomic lung resection for operable non-small cell carcinoma	25 (13/12)	No statistically significant difference (age, sex, operative time, and duration of chest-tube drainage)	(A) EA	(B) Sham		(1) VAS (2) PCA morphine usage (3) Peak flow rate (4) chest drain duration (5) details of postoperative complications

Abbreviations: EA = electroacupuncture, PCIA = patient-controlled intravenous analgesia, VAS = visual analogue scale, β-EP = beta endorphin, 5-HT = 5-hydroxytryptamine, PGE_2_ = prostaglandin E2, SAS = sedation-agitation scale, ICU = intensive care unit, EAS = electroacupuncture stimulation, PVB = paravertebral block, PCA = patient controlled analgesia, TENS = transcutaneous electrical nerve stimulation, EAA = electroacupuncture-anesthesia, MV = mechanical ventilation, NAS = numerical analogue scale.

Group label A and B in the sample size category matches with the names in the intervention category. Some studies contain more than 2 groups.

In Zhou (2017) [22], 60 patients undergoing elective radical resection to treat esophageal cancer were treated using either EA or patient-controlled intravenous anesthesia (PCIA) only, with 30 patients in each group. The VAS scores 2, 12, and 24 hours after surgery were all significantly lower in the EA group than in the control group (p < 0.05 in all cases). The VAS score 24 hours after surgery was 2.46 ± 0.78 in the EA group, while that in the control group was 3.39 ± 0.76. The total dose of sufentanil in the EA group was 1.83 ± 0.56 mg, which was statistically lower than the 2.54 ± 0.64 mg in the control group (p < 0.05).

In Chen (2016) [5], 92 patients undergoing small incision lobectomy surgery were treated using either EA or sham acupuncture, with 46 patients in each group. Significant differences in VAS scores between the two groups were observed 2, 24, 48, and 72 hours after surgery (F_(1360)_ = 30.54; p < 0.001). That is, the cumulative VAS scores were significantly lower in the EA group than in the sham group during the test period (p < 0.001). The total dose of supplementary sauteralgyl was 9.2 ± 2.8 mg/kg in the EA group and 11.5 ± 1.8 mg/kg in the sham group (p < 0.0001), constituting a 20% reduction in the EA group.

In Yu (2016) [24], 50 patients undergoing cardiac surgery were treated using either acupuncture combined with medicine (ACM) or CA using injected dexmedetomidine. The static and dynamic VAS scores did not differ significantly (p > 0.05). The injection dosage of dexmedetomidine and morphine hydrochloride was significantly lower in the ACM group (p < 0.05). The dosage of morphine hydrochloride was 11.20 ± 6.20 in the ACM group and 15.28 ± 6.78 in the CA group.

In Wang (2015) [25], 80 patients undergoing elective lobectomy were treated with either EA-assisted general anesthesia or general anesthesia alone, with 40 patients in each group. Significant differences in VAS scores were observed at 0.5, 1, 2, 4, 12, 24, and 48 hours after surgery (p < 0.05 in all cases). The total dosage of sufentanil was 123.4 ± 5.7 μg in the EA group and 142.5 ± 6.2 μg in the control group, which constituted a significantly lower dosage in the EA group (p = 0.02). Fewer patients in the EA group required dezocine for pain management after surgery (3 patients; 7.5%) than in the control group (12 patients; 30%) (p = 0.01).

In Xie (2014) [26], 60 patients who underwent radical esophagectomy to treat cancer were treated using EA, sham acupuncture (sham), or general anesthesia only (control), with 20 patients in each group. Patients in the EA group had the lowest VAS scores after surgery (p < 0.05). The mean total dose of sufentanil was 115 ± 6.0 μg in the EA group, which was significantly lower than in the control group (134.3 ± 5.9 μg) and sham (133.5 ± 7.0 μg) groups (p < 0.05).

In Yu (2013) [26], 40 patients undergoing unilateral lobectomy were treated with either EA combined with paravertebral block (PVB) or just PVB, with 20 patients in each group. The static VAS scores at 12, 24, and 36 hours, and the dynamic VAS scores at 12 and 35 hours after surgery in the EA+PVB group were significantly lower than in the PVB group (p < 0.05 in all cases). The intraoperative dosage of remifentanil and the dosage of sufentanil via PCIA were significantly lower in the EA+PVB group (67.3 ± 11.3 μg) than in the PVB group (82.2 ± 9.9 μg) (p < 0.01).

In Zhao (2013) [28], 120 patients undergoing thoracic surgery were treated using EA or PCIA, with 60 patients in each group. The VAS scores at every measured time point, including 24 hours later, were lower in the EA group than in the medication group (p < 0.01). The VAS scores 24 hours after surgery were 1.55 ± 0.62 and 2.01 ± 0.73, respectively. The study compared VAS, analgesia effect, safety, and β-endorphins, but did not compare the total dose of analgesics.

In Coura (2011) [29], 22 patients undergoing conventional elective heart surgery were treated using either preoperative EA at bilateral points or sham transcutaneous electrical nerve stimulation, with 13 and nine patients in each group, respectively. The average total doses of fentanyl given were 13.1 ± 2.2 μg/kg and 16.3 ± 1.6 μg/kg in the treatment and control groups, respectively, constituting a significantly lower value in the treatment group (p < 0.002). The doses of PCIA were 4.1 ± 2.0 μg/kg and 6.9 ± 1.7 μg/kg in the treatment and control groups respectively, constituting a significantly lower value in the treatment group (p < 0.003).

In Zhu (2011) [30], 120 patients undergoing lung-cancer thoracotomy were randomly allocated into four groups, with 30 patients in each group. Group A was treated using pre-, intra-, and postoperative acupuncture analgesia combined with PCIA. Group B was treated using pre- and intraoperative acupuncture analgesia combined with PCIA. Group C was treated using postoperative acupuncture analgesia combined with PCIA. Group D was treated using PCIA only. A one-way ANOVA test on postoperative fentanyl use in the four groups gave a p-value of < 0.05, indicating a significant difference. This study showed that acupuncture can produce remarkable effects in terms of postoperative analgesia, even being associated with 20% lower fentanyl PCIA.

In Bachleda (2010) [31], 34 patients undergoing primary elective coronary artery bypass graft were treated with either EA or conventional opioid-analgesia only, with 16 and 18 patients in each group, respectively. The numerical analogue scale score in the EA group was 1.8 ± 1.1, which was significantly lower than in the opioid group (4.5 ± 2.3) (p = 0.001). Sufentanil consumption in the EA group was 46 ± 13 μg, which was significantly lower than in the opioid group (354 ± 71 μg) (p ≤ 0.001).

In Wong (2006) [5], 25 patients with operable non-small cell carcinoma who underwent thoracotomy were treated using either EA or sham acupuncture in addition to routine oral analgesics and PCIA for postoperative pain control, with 13 and 12 patients in each group, respectively. Patients in the EA group tended to have lower VAS scores between postoperative days 2 and 6, although this did not reach significance. The cumulative dose of PCIA morphine used on postoperative day 2 was significantly lower in the EA group (7.5 ± 5 mg vs. 15.6 ± 12 mg; p < 0.05).

### Intervention

PC4, PC6, TE6, and LI4 were commonly selected acupuncture points. Lower frequencies were used more than higher frequencies (Table 2).

**Table 2:** Summary of acupuncture points used in the intervention group.

Author (year)	Frequency (Hz)	Acupuncture points	Rationale for selection
**Zhou (2017)** [22]	4~100	Neimadian (Extra), PC6	Previous studies
**Chen (2016)** [23]	2	LR 3, GB34, TE5, LU5	TCM meridian theory
**Yu (2016)** [24]	5	GV24, EX-HN3	Records on acupuncture
**Wang (2015)** [25]	n.r.	PC6, PC3, PC4	n.r.
**Xie (2014)** [26]	2 and 20	PC4, PC6	previous studies
**Yu (2013)** [27]	2/100 (AM)	PC6, TE5, TE6, LI4	n.r.
**Zhao (2013)** [28]	2~17	Neimadian (Extra), PC6	Previous studies
**Coura (2011)** [29]	3/15	LI4, LI11, LR3, ST36, PC6, TE5 (b)	n.r.
**Zhu (2011)** [30]	4–8	SI3, TE6, PC6, LI4 (b)	n.r.
**Bachelda (2010)** [31]	15–30	TE9, ST10, heart (100) to trachea (103) ear point	n.r.
**Wong (2006)** [5]	60	LI4, GB34, GB36, TE8 (b)	n.r.

Abbreviations: n.r.: Not reported, b: Bilateral.

#### Outcome measures

Ten of the eleven studies used a pain score to measure analgesic effects [5,22–29,31]. VAS was the most used pain scale in the studies, with eight of the ten studies using it. The total dose of opioids was reported in ten studies [5,22–27,29–31]. The type of opioid differed among the studies, although sufentanil was prevalent among them. Five studies selected sufentanil for intravenous analgesia. Several studies have also measured the amount of released chemicals with analgesic effects, such as β-endorphin and 5-Hydroxytryptamine. The incidence of postoperative complications has also been reported in some studies.

### Risk of bias in included studies

**Fig 2:**
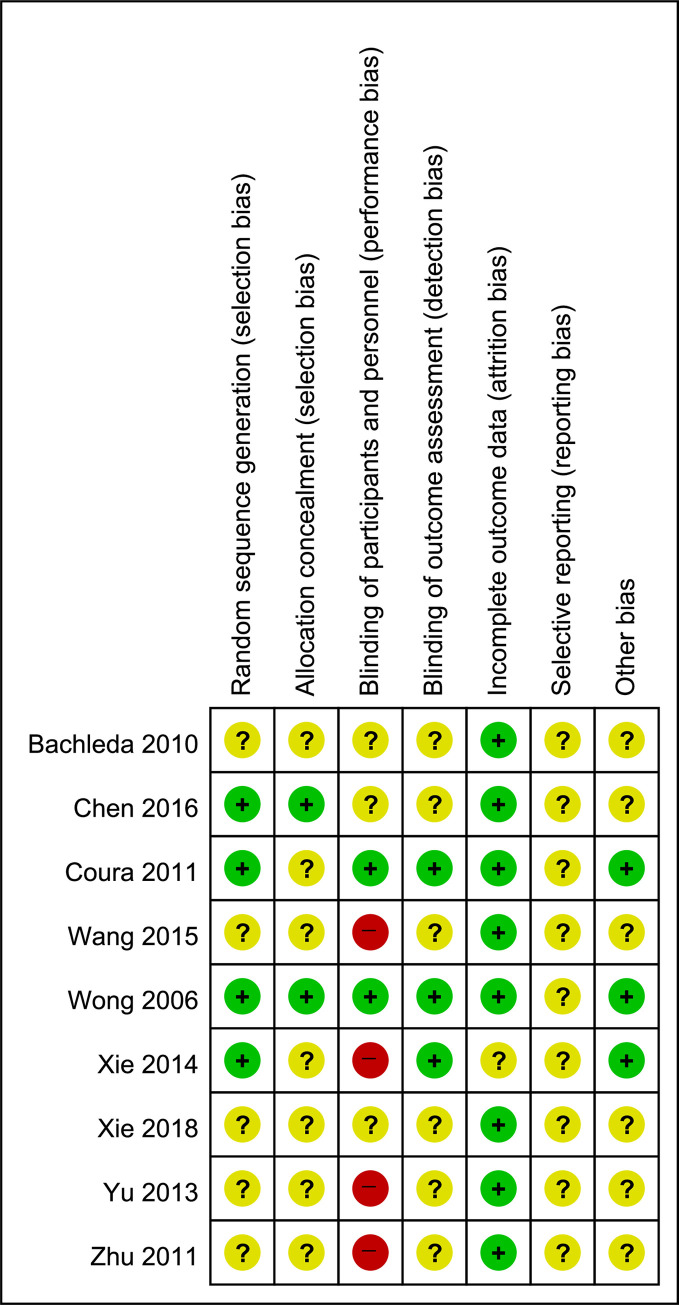
Risk of bias summary.

**Fig 3:**
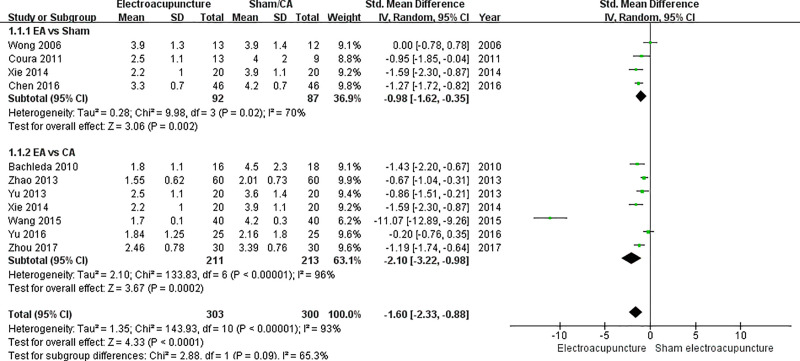
Forest plot of pain score 24 hours after surgery before sensitivity analysis.

As shown in Figs 2 and 3, the Cochrane risk of bias tool was used to assess the risk of bias and study quality of the 11 included studies. Among the 11 studies, Wong (2006) [5] and Coura (2011) [29] were evaluated as having lower risk of bias than the others.

Six of the 11 studies used a random sequence or a list generated by computers. Thus, they were evaluated as “low-risk” in the random sequence generation section. In the allocation concealment section, only two studies used an allocation process involving sealed envelopes; they were evaluated to be at “low risk.”

With regards to blinding of participants and personnel, it is impossible for personnel performing EA to be blinded to the intervention. However, in Coura (2011) [29] and Wong (2006) [5], the studies were successfully double-blinded, because the practitioners performing EA did not take part in postoperative patient care or data collection. The patients were blinded in four studies, including the two that had sham groups as control groups. However, only the two studies that mentioned double-blinding were evaluated as “low-risk.” Others were evaluated as “unclear-risk” at least. Seven studies were evaluated as “high-risk” since they had CA groups as controls, so it was impossible to successfully blind due to the nature of the intervention.

With regards to blinding of outcome assessment, three studies were evaluated as “low-risk,” because the outcome assessors were successfully blinded.

Concerning incomplete outcome data, all studies except one were evaluated as “low-risk,” since all participants completed every outcome assessment, except for patients who were unable to be assessed due to emergency. In Xie (2014) [26], it was unclear whether all participants completed any outcome assessment other than the total dose of sufentanil, patient demand, and rate of pain rescue using dezocine.

In the selective reporting section, all studies were evaluated as “unclear-risk” because, without a trial protocol, it was unclear based on the results whether any outcomes were measured but not reported.

In the other potential sources of bias section, the risk of bias was assessed based on the amount of information about bias control.

### Quality of evidence

The strength of the body of evidence was evaluated using the GRADE guidelines (Table 3). This assessment was performed on the group of studies that were quantitatively synthesized, which were grouped according to primary and secondary outcome measures. Other than in studies that compared pain score between EA and Sham groups, the certainty of cumulative evidence was low in all studies. Low ratings were given because there were inconsistencies and high risk of bias in all studies. The risk of bias in each group was rated as “not serious” if the number of studies with a high risk of bias was less than 25% of the total. If the risk was between 25% and 50%, it was rated as “serious”; if it was more than 50%, it was considered “very serious.” Inconsistency was rated as “very serious” if the heterogeneity was higher than 75%; this was the case in all but one group. Low quality of evidence indicates that some RCTs included in the present study contained flaws that could have affected the aim of our study.

**Table 3:** GRADE evidence table.

Certainty assessment							№ of patients		Effect	Certainty	Importance
№ of studies	Study design	Risk of bias	Inconsistency	Indirectness	Imprecision	Other considerations	Electro acupuncture	Control	Absolute (95% CI)		
**Pain score**											
10	randomised trials	serious ^a^	very serious ^b^	not serious	not serious	none	303	300	SMD **1.6 lower** (2.33 lower to 0.88 lower)	⨁◯◯◯ VERY LOW	CRITICAL
**Pain score—EA vs Sham**											
4	randomised trials	not serious	serious ^c^	not serious	not serious	none	92	87	SMD **0.98 lower** (1.62 lower to 0.35 lower)	⨁⨁⨁◯ MODERATE	CRITICAL
**Pain score—EA vs CA**											
7	randomised trials	serious ^d^	very serious ^e^	not serious	not serious	none	211	213	SMD **2.1 lower** (3.22 lower to 0.98 lower)	⨁◯◯◯ VERY LOW	CRITICAL
**Total dose of analgesics**											
10	randomised trials	serious ^f^	very serious ^g^	not serious	not serious	none	273	270	SMD **1.74 lower** (2.35 lower to 1.14 lower)	⨁◯◯◯ VERY LOW	CRITICAL
**Total dose of analgesics—EA vs Sham**											
4	randomised trials	not serious	very serious ^h^	not serious	not serious	none	92	87	SMD **1.39 lower** (2.25 lower to 0.53 lower)	⨁⨁◯◯ LOW	CRITICAL
**Total dose of analgesics—EA vs CA**											
7	randomised trials	very serious ^i^	very serious^j^	not serious	not serious	none	181	183	SMD **1.96 lower** (2.82 lower to 1.1 lower)	⨁◯◯◯ VERY LOW	CRITICAL

**Abbreviation CI:** Confidence interval; **SMD:** Standardised mean difference.

a. Three out of ten studies had a high risk of bias.

b. Higgins heterogeneity test score (I squared) is 93%.

c. Higgins heterogeneity test score (I squared) is 70%.

d. Three out of seven studies had a high risk of bias.

e. Higgins heterogeneity test score (I squared) is 96%.

f. Four out of ten studies had a high risk of bias.

g. Higgins heterogeneity test score (I squared) is 88%.

h. Higgins heterogeneity test score (I squared) is 81%.

i. Four out of seven studies had a high risk of bias.

j. Higgins heterogeneity test score (I squared) is 91%.

### Meta-analysis

Ten studies were included in the data synthesis of pain score 24 hours after the operation [5,22–29,31]. Since Xie (2014) had both sham acupuncture group and CA groups as control groups, providing two sets of data in the study, a total of eleven data sets were synthesized [26]. In the subgroup EA vs. sham acupuncture, the SMD with 95% CI was -0.98 (-1.62 to -0.35) (p = 0.002). In the subgroup EA vs. CA, the SMD with 95% CI was -2.10 (-3.22 to -0.98) (p = 0.0002). When all eleven data sets were synthesized, the SMD with 95% CI was -1.60 (-2.33 to -0.98) (p < 0.0001) (Fig 3).

**Fig 4:**
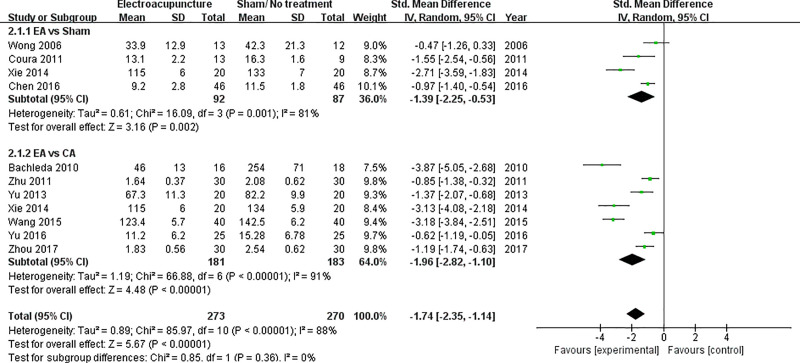
Forest plot of total dose of opioid analgesics before sensitivity analysis.

Ten studies were included to analyze the total dose of analgesics [5,22–27,29–31]. Since Xie (2014) also measured the total dose of analgesics, a total of eleven data sets were synthesized [26]. In the subgroup EA vs. sham acupuncture, the SMD with 95% CI was -1.39 (-2.25 to -0.53) (p = 0.002). In the subgroup EA vs. CA, the SMD with 95% CI was -1.96 (-2.82 to -1.10) (p < 0.00001). In the group including all eleven data sets, the SMD with 95% CI was -1.74 (-2.35 to -1.14) (p < 0.00001) (Fig 4).

**Fig 5:**
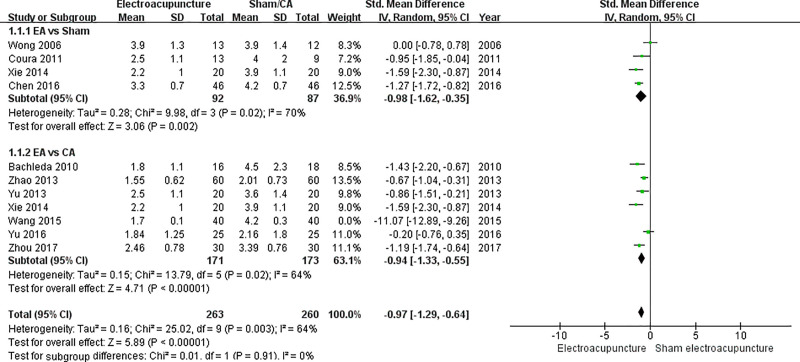
Final forest plot of pain score 24 hours after sensitivity analysis.

A sensitivity analysis was performed to determine the precision of methodology. According to the analysis, using the random-effect model and calculating SMD were the most appropriate methodology for the meta-analysis. In the subgroup EA vs CA of pain score 24 hours after surgery, Wang (2015) [25] caused severe heterogeneity with noticeably large SMD value [25]. When Wang (2015) [25] was eliminated as a subject, the I^2^ estimate decreased from 96% to 64%. Therefore, the final meta-analysis included only nine outcomes of pain score 24 hours after surgery, excluding Wang (2015) [25] (Fig 5).

**Fig 6:**
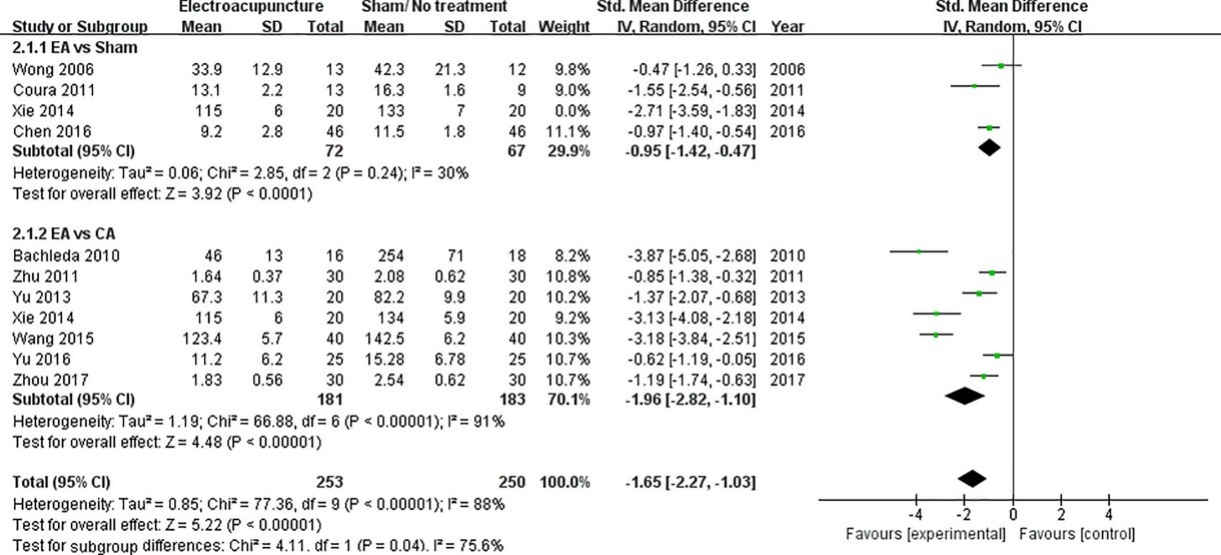
Final forest plot of total dose of opioid analgesics after sensitivity analysis.

In the subgroup EA vs sham of total dose of opioid analgesics, Xie (2014) caused severe heterogeneity [26]. When Xie (2014) [26] was removed as a subject, the I^2^ estimate decreased significantly from 81% to 30%. Therefore, the final meta-analysis excluded total dose of opioid analgesics of EA vs sham acupuncture in Xie (2014) [26] (Fig 6).

### Safety

Wong (2006) reported that no mortality, complications, or adverse effects occurred in either the EA or sham acupuncture groups [5]. The other ten studies did not mention any information on adverse effects.

## Discussion

EA alleviates pain by inducing release of bioactive chemicals that block pain through peripheral, spinal, and supraspinal mechanisms [8]. Released endogenous opioids plays a major role by desensitizing peripheral nociceptors, decrease cytokines in inflammatory sites, and descending inhibitory system [8]. Besides opioids, various signal molecules such as cholecystokinin octapeptide, norepinephrine, 5-hydroxytryptamine and glutamate also take part in EA analgesia [11].

The objective of the present study was to evaluate the analgesic effects of EA in patients who had undergone thoracotomies by reviewing and quantitively synthesizing data from previous studies. Nine databases were searched using keywords relevant to the framed PICO, and eleven studies that met the pre-set inclusion criteria were included in the systematic review and meta-analysis, after a three-step exclusion process. The outcome measures selected for the meta-analysis were pain score 24 hours after surgery and total opioid analgesic dose, because these are the most common measures of analgesic effect in studies on pain management.

The first outcome measure of the meta-analysis was pain score 24 hours after surgery. Ten RCTs were included in the initial analysis of the outcome measure but one RCT was excluded after the sensitivity analysis. The result showed that pain score 24 hours after thoracotomy was significantly lower in the EA group than in both control groups. The second outcome measure was the amount of opioid use and ten RCTs were included initially for the analysis. After the sensitivity analysis, Xie (2014) [26] was excluded in the subgroup EA vs sham acupuncture. Since Xie (2014) [26] provided the comparison with both control groups, it was still included in the subgroup EA vs CA. The result of the final meta-analysis showed that the total dose of opioid analgesics was significantly lower in the EA group than in the control groups. Therefore, our systematic review indicated that EA might be effective in controlling pain after thoracotomy.

There were several limitations in the present systematic review and meta-analysis that should be considered. Firstly, in terms of quality assessment, the overall quality of the included studies was not high. Only two studies were evaluated as having low risk in more than half of the sections in the risk of bias tool. Several studies did not mention many of the efforts made to reduce biases, including randomization. Secondly, most of the included RCTs were performed in China, so the research had low generalizability. Thirdly, the second outcome measure was total dose of opioid analgesics, and four different opioids were used in the included studies: sufentanil, fentanyl, sauteralgyl, and morphine (Table 1). This could cause high heterogeneity between the collected data. The heterogeneity in the subgroup EA vs CA of total dose of opioid analgesics was high. Subgroup analysis and sensitivity analysis were performed to adjust for heterogeneity, which led to decrease of heterogeneity in other subgroups. However, heterogeneity was not due to a single study inclusion in this subgroup. If more studies are published in the future that focus on EA, more specific meta-analysis could be performed according to the type of opioid. Also, subgroup analysis according to the type of surgery or the acupuncture points used in the EA treatment could be performed if data can be collected enough. Fourthly, publication bias was not assessed. It was planned in the protocol to use a funnel plot to assess publication bias if more than 10 RCTs were included in the meta-analysis. Since only ten studies were included for both outcome measures in the meta-analysis, it was decided that the number of studies was not enough to assess the publication bias [32,33]. When the systematic review and meta-analysis is updated with future studies publication bias should be evaluated.

The analgesic effect of EA has been confirmed both empirically and scientifically, so further rigorously designed RCTs should be carried out to overcome the limitations of the present study and reinforce the evidence that EA is effective in treating post-thoracotomy pain.

Furthermore, almost 50% of patients who have undergone thoracotomy suffer from chronic post-thoracotomy pain a year after the surgery [34]. Chronic post-thoracotomy pain (post-thoracotomy syndrome or post-thoracotomy neuralgia) is defined by the International Association for the Study of Pain as “pain that recurs or persists along a thoracotomy incision at least 2 months following the surgical procedure” [35]. Even though post-thoracotomy pain commonly lingers in the long term, a limited number of studies have evaluated the effect of EA on patients with chronic post-thoracotomy pain. We hope that several high-quality studies covering EA’s effect on chronic post-thoracotomy pain will be also performed in the future. If it is also found to be effective on chronic pain, EA could contribute more widely to the postoperative pain management of patients undergoing thoracotomy.

## Conclusion

In conclusion, according to the synthesized data of previous studies, EA showed the possibility of reducing pain for patients after thoracotomy with less amount of opioid analgesics. However, the meta-analysis had limitations, namely the low quality and heterogeneity of the included studies. A sensitivity analysis and subgroup analysis were performed to adjust the heterogeneity, but they were not enough. To overcome these limitations, several rigorously designed RCTs should be carried out to confirm EA’s efficacy and safety before translating into clinical practice.

## Supporting information

S1 Table PRISMA checklist. (DOC)

S2 Table Search strategy for MEDLINE. TI: title, Ab: abstract.
(DOCX)
