# Supplementary material for: Electroacupuncture for post-thoracotomy pain: A systematic review and meta-analysis
Source: PLoS One. 2021 Jul 7;16(7):e0254093. doi: 10.1371/journal.pone.0254093 (PMC8263274; doi:10.1371/journal.pone.0254093)
Supplement: S2 Table — TI: title, Ab: abstract. (DOCX) [file pone.0254093.s002.docx]

S2 Table. Search strategy for MEDLINE

| No | Search Terms |
| --- | --- |
| 1 | TI "Thoracotomy" OR AB"Thoracotomy" |
| 2 | TI"Thoracic Surgery" OR AB"Thoracic Surgery" |
| 3 | TI"Lobectomy" OR AB"Lobectomy" |
| 4 | TI"Pneumonectomy" OR AB"Pneumonectomy" |
| 5 | TI"Pneumectomy" OR AB"Pneumectomy" |
| 6 | TI"Esophagectomy" OR AB"Esophagectomy" |
| 7 | TI"Open heart surgery" OR AB"Open heart surgery" |
| 8 | TI"Cardiac surgery" OR AB"Cardiac surgery" |
| 9 | OR 1-8 |
| 10 | TI"Video-assisted thoracic surgery" |
| 11 | TI"Pain" OR AB"Pain" |
| 12 | TI"Postoperative" OR AB"Postoperative" |
| 13 | TI"Perioperative" OR AB"Perioperative" |
| 14 | TI"analgesia" OR AB"analgesia" |
| 15 | TI"analges*" OR AB"analges*" |
| 16 | OR 10-15 |
| 17 | TI"acupuncture" OR AB"acupuncture" |
| 18 | TI"acupressure" OR AB"acupressure" |
| 19 | TI"acupoint" OR "acupoint" |
| 20 | TI"acup*" OR "acup*" |
| 21 | TI"electroacupuncture" OR "electroacupuncture" |
| 22 | OR 17-21 |
| 23 | (#9 NOT #10) AND #16 AND #22 |
